# Promoting Connections Through Creative Approaches to Research Engagement for Older African Americans

**DOI:** 10.1093/geroni/igab046.1178

**Published:** 2021-12-17

**Authors:** Tam Perry, Jamie Mitchell, Kent Key, Vanessa Rorai, Sean Knurek, Peter Lichtenberg

**Affiliations:** 1 Wayne State University, Detroit, Michigan, United States; 2 University of Michigan School of Social Work, Ann Arbor, Michigan, United States; 3 Michigan State University, Flint, Michigan, United States; 4 Wayne State University Institute of Gerontology, Detroit, Michigan, United States; 5 Michigan State University, Corunna, Michigan, United States

## Abstract

This presentation will feature innovative retention approaches that contributed to sustaining connections to older Black participants in the long-standing Healthier Black Elders Center (HBEC). The HBEC aims to address and reduce health disparities through research and education. In 2020, this outreach has included a telephone outreach program and a weekly social group, “The Party Line,” to promote connections and collect data on mental health, coping mechanisms and newly acquired skills, as well as health care access including access to masks, testing and tele-health. The presentation will also describe tailored approaches to initiating a Community Advisory Board and programming in Flint, MI and creative efforts to retain participants in Detroit, MI, thus ensuring the relationships between researchers and older community members are sustained despite program modifications.

